# Toxicodynamic insights of 2C and NBOMe drugs – Is there abuse potential?

**DOI:** 10.1016/j.toxrep.2025.101890

**Published:** 2025-01-03

**Authors:** Eva Gil-Martins, Daniel José Barbosa, Fernanda Borges, Fernando Remião, Renata Silva

**Affiliations:** a Associate Laboratory i4HB-Institute for Health and Bioeconomy, Faculty of Pharmacy, University of Porto, Porto, Portugal; b UCIBIO-Applied Molecular Biosciences Unit, Laboratory of Toxicology, Department of Biological Sciences, Faculty of Pharmacy, University of Porto, Porto, Portugal; c CIQUP-IMS/Department of Chemistry and Biochemistry, Faculty of Sciences, University of Porto, Porto, Portugal; d Associate Laboratory i4HB - Institute for Health and Bioeconomy, University Institute of Health Sciences - CESPU, Gandra, Portugal; e UCIBIO - Applied Molecular Biosciences Unit, Translational Toxicology Research Laboratory, University Institute of Health Sciences (1H-TOXRUN, IUCS-CESPU), Gandra, Portugal; f i3S - Instituto de Investigação e Inovação em Saúde, Universidade do Porto, Porto, Portugal

**Keywords:** New psychoactive substances, Psychedelic phenethylamines, 2C drugs, NBOMe drugs, Abuse potential

## Abstract

Drug use represents a prevalent and multifaceted societal problem, with profound implications for public health, social welfare, and economic stability. To circumvent strict international drug control regulations, there is a growing trend in the development and market introduction of novel psychoactive substances (NPS), encompassing a wide range of compounds with psychoactive properties. This includes, among other classes of drugs, the phenethylamines. Originally derived from natural sources, these drugs have garnered particular attention due to their psychedelic effects. They comprise a broad spectrum of compounds, including 2,5-dimethoxyphenylethylamine (2C) drugs and their corresponding *N*-(2,5-dimethoxybenzyl)phenethylamine (NBOMe). Psychedelics are conventionally perceived as having low addiction potential, although recent reports have raised concerns regarding this topic. These substances primarily interact with serotonin receptors, particularly the 5-HT_2A_ subtype, resulting in alterations in sensory perception, mood, and introspective experiences. In addition to their psychedelic properties, 2C and NBOMe drugs have been associated with a multitude of adverse effects, such as cardiovascular complications and neurotoxicity. This manuscript provides a comprehensive review of the psychedelic pathways underlying 2C and NBOMe designer drugs, focusing on their interactions with serotonergic and other neurotransmitter systems, shedding light on their potential for abuse.

## Highlights

• Psychedelic effects are mainly driven by their interaction with serotonin (5-HT) receptors
• The addition of an *N*-benzyl moiety enhances drug strength, especially 5-HT_2a_ affinity
• 2C and NBOMe drugs trigger *in vivo* 5-HT_2a_-mediated behaviors
• 2 C and NBOMe drugs induce rewarding and reinforcing effects via dopaminergic mechanisms

## Graphical Abstract

**Figure:**
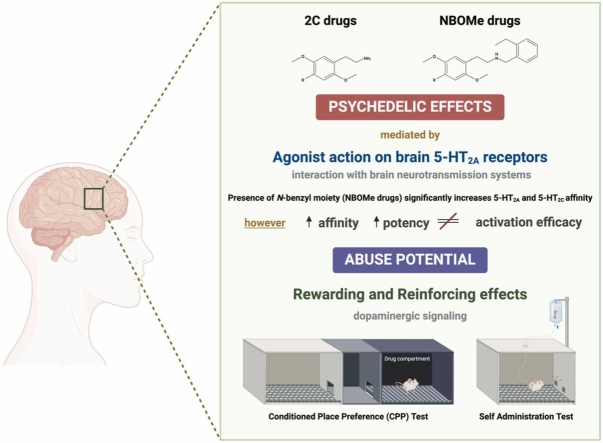

## 1 Introduction

**Fig. 1:**
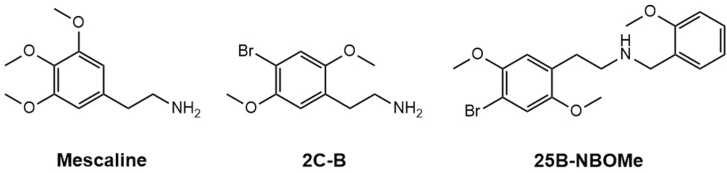
Chemical structures of mescaline, 2C-B and 25B-NBOMe. The presence of different substituents (functional groups or halogens) at position-4 of the aromatic ring leads to the formation of distinct drugs (if the substituent is a methyl group the drug is known as 2C-D and 25D-NBOMe, respectively; if the substituent is an iodine, the drug is known as 2C-I and 25I-NBOMe, respectively).

The world of new psychoactive substances (NPS) includes a vast array of compounds. Among these are the 2C and NBOMe drugs (Fig. 1). These drugs are part of the substituted phenethylamine class and became popular for their potent psychedelic properties, often described as producing strong visual and sensorial experiences [1]. 2C and NBOMe drugs mainly act by interacting with serotonin (5-HT) receptors in the brain, especially the 5-HT_2A_ receptor subtype, which is generally regarded as central to their psychedelic properties [2], [3].

Alexander Shulgin originally synthesized the 2C series of drugs in the 1970s and 1980s [4]. These compounds are structurally related to mescaline (Fig. 1), a naturally present psychedelic phenethylamine in several species of cacti [5]. 2C drugs feature two methoxy groups at the 2- and 5-positions of the aromatic ring, along with diverse substituents at the 4-position [4]. For example, if the substituent is a bromine, the resulting compound is known as 2C-B (Fig. 1). NBOMe drugs represent a newer class of synthetic psychedelics, created by adding an *N*-2-methoxybenzyl group to the molecular structure of the 2C series of drugs [6]. Similar to 2C drugs, NBOMe drugs can also have various substitutions at the 4-position of the aromatic ring. For instance, if the substituent is a bromine, the resulting compound is known as 25B-NBOMe (Fig. 1).

NBOMe drugs are generally more potent in inducing psychedelic effects than 2C drugs. While both types of drugs carry risks, NBOMe compounds are linked to a greater incidence of negative side effects and fatalities [7], [8].

Traditionally, psychedelics have been viewed as having low potential for addiction, not causing cravings or compulsive drug-seeking behavior [1]. However, the activation of the brain’s reward system and the potential for addition of 2C and NBOMe drugs have already been reported [9], [10], [11], [12], [13], [14], [15].

Understanding these drugs’ toxicodynamic properties will help to elucidate how they affect neurotransmitter systems and brain function, which can provide insights into their potential for abuse. Thus, this review focus on the toxicodynamic aspects of 2C and NBOMe drugs, exploring their potential for abuse.

## 2 Toxicodynamic of 2C and NBOMe based drugs

**Fig. 2:**
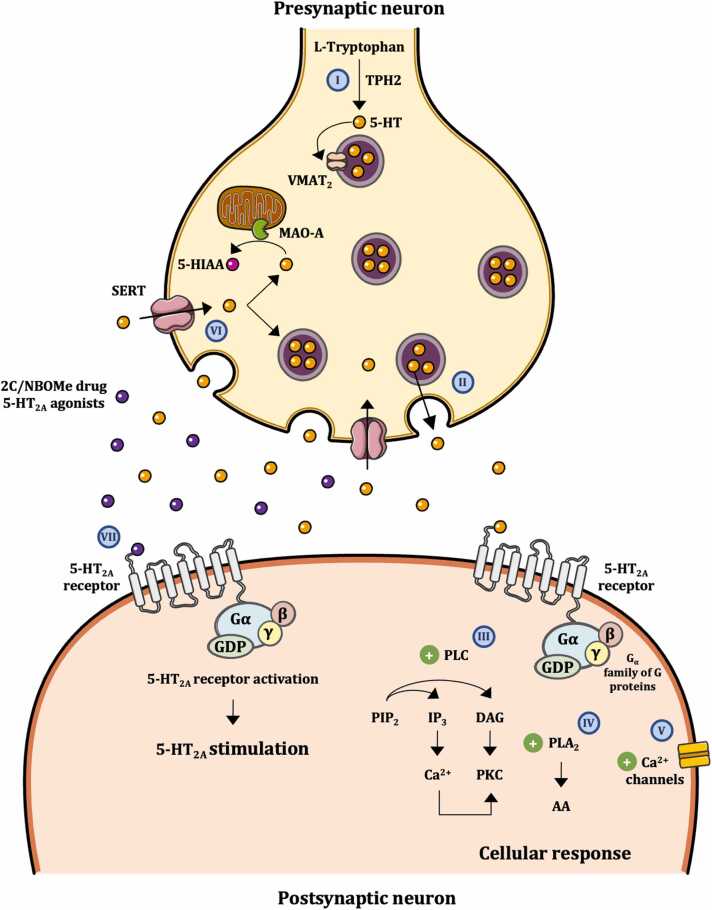
Mechanism of action of 2C and NBOMe drugs in serotoninergic neurons. (I) Serotonin (5-HT) is synthetized from L-Tryptophan by tryptophan hydroxylase 2 (TPH_2_). When inside the presynaptic neuron, 5-HT is stored in neurotransmitter vesicles via the vesicular monoamine transporter (VMAT_2_). (II) Once released, 5-HT can modulate postsynaptic neurotransmission by binding to Gα-coupled 5-HT_2A_ receptors, that can: (III) activate phospholipase C (PLC), originating the hydrolysis of phosphatidylinositol 4,5-bisphosphate (PIP_2_) into inositol 1,4,5-trisphosphate (IP_3_; leads to Ca^2+^ release from the endoplasmic reticulum) and diacylglycerol (DAG; stimulates protein kinase C (PKC) activity); (IV) activate phospholipase A2 (PLA_2_), stimulating arachidonic acid (AA) release, through complex signaling mechanism involving Gα_i/o_ and G_12/13_ proteins; and/or (V) couple to Ca^2+^ channels. (VI) The 5-HT transporter (SERT) reuptakes 5-HT back to the presynaptic neuron, where it can be restored into vesicles or degraded by monoamine oxidase (MAO) A to 5-hydroxyindole amino acid (5-HIIA). (VII) 2C and NBOMe drugs act as 5-HT_2A_ receptor agonists. The stimulation of these receptors mediates the characteristic psychedelic effects of these drugs.

The effects of psychedelic substances are primarily driven by their interaction with 5-HT receptors (Fig. 2) [1]. 5-HT performs a multitude of roles in human physiology, influencing systems such as pulmonary, cardiovascular, gastrointestinal and central nervous systems, among others. It exerts its effects through fourteen receptor subtypes, which are categorized into seven families (5-HT_1_ - 5-HT_7_), of which thirteen are G protein coupled receptors (GPCR) and one (5-HT_3_) is a ligand-gated ion channel [16].

5-HT_2_ receptors are primarily associated with the Gα_q_ (Gα_q/11_) family of G proteins, which activate phospholipase C (PLC). This initiates the hydrolysis of phosphatidylinositol 4,5-bisphosphate (PIP_2_) into inositol 1,4,5-trisphosphate (IP_3_) and diacylglycerol (DAG). IP_3_ mediates the release of Ca^2+^ from intracellular stores, such as the endoplasmic reticulum, while DAG induces protein kinase C (PKC) activity. Furthermore, these receptors can also function via phospholipase A2 (PLA_2_), which stimulates arachidonic acid (AA) release (Fig. 2), a process that involves other G-protein mediated signaling pathways, including the activation of Gα_i/o_ proteins [17], [18], [19]. 5-HT_2A_ receptors can also couple to Ca^2+^ channels to modulate various cellular processes [17], [20]. Conversely, 5-HT_1_ receptors are coupled to the Gα_i/__o_ family of G proteins, which inhibit adenylyl cyclase (AC), ensuing in a reduction of intracellular cyclic adenosine monophosphate (cAMP) [21].

Currently, it is generally accepted that a full or partial agonism on 5-HT_2A_ receptors (Fig. 2) mediates the molecular mechanisms key to the effects of classic psychedelics. However, it is likely that these phenethylamines derivatives are not selective for 5-HT_2A_ receptors alone, and other central nervous system receptors may also impact their overall effects. In fact, 5-HT_2B_, 5-HT_2C_ and 5-HT_1A_ have also been proposed to contribute to the mechanism of action of several psychedelic phenethylamines [2], [3], [22].

### 2.1 In vitro studies

#### 2.1.1 Affinity, potency, efficacy and selectivity for 5-HT receptors

In general, the binding affinity of psychedelic phenethylamines for 5-HT_2_ receptors significantly increases with the molecular structures that include an *N*-benzyl moiety [23], [24], [25], [26].

Rickli et al. investigated, *in vitro,* the receptor binding profile of a series of 2C drugs – including 2C-B, 2C-C, 2C-D, 2C-E, 2C-H, 2C-I, 2C-N, 2C-P, 2C-T-2, 2C-T-4, 2C-T-7, and mescaline – and their NBOMe equivalents (LSD was included as positive control) [25]. The inclusion of a *N*-2-methoxybenzyl group (NBOMe drugs) leads to an increase in the already high affinity for 5-HT_2A_ receptors compared to their 2C parent drugs, with all NBOMe drugs displaying high affinity for this receptor even at low nanomolar or sub-nanomolar concentrations (Table 1). Nonetheless, relative to 2C drugs, the activation efficacy of NBOMe drugs was reduced, even with increased activation potency, suggesting that an increased affinity for 5-HT_2A_ receptor does not directly correlates with drug-mediated activation efficacy (Table 1). Consistently, the addition of a *N*-2-methoxybenzyl substituent improved the binding affinity for 5-HT_2C_ receptors (Table 1), while preserving the low selectivity for 5-HT_2A_ relative to 5-HT_2C_ receptors. On the contrary, the binding affinity for 5-HT_1A_ receptors was reduced for the NBOMe drugs when compared to their analogs (Table 1) with a significant increase in the selectivity for 5-HT_2A_ over 5-HT_1A_ receptors. Moreover, although *N*-2-methoxybenzyl replacement augmented 5-HT_2B_ activation potency, it reduced the receptor activation efficacy (Table 1) [25]. Similarly, Elmore et al. explored the neuropharmacological effects of 2C-C and 2C-I in comparison to the effects elicited by their NBOMe counterparts. In brain tissue of Sprague-Dawley rats, 25C-NBOMe and 25I-NBOMe exhibited a 35- and 32-fold higher affinity for 5-HT_2A_ receptors, respectively, when compared to their 2C analogs. As previously outlined, in the functional assays (Ca^2+^ mobilization in HEK 293 cells transfected with human 5-HT_2A_ or 5-HT_2C_) the NBOMe drugs were considerably less potent than 2C drugs – all tested drugs were 5-HT_2A_ full agonists (Table 2), with 2C-C and 2C-I being 13-fold and 8-fold more potent than 25C-NBOMe and 25I-NBOMe, respectively. In the 5-HT_2C_ functional assay, drugs exhibited similar potencies (EC_50_ ranging from 47.2 to 178 nM), but the NBOMe drugs were more effective than their 2C analogs (maximum effect of: 2C-C = 29 % *vs* 25C-NBOMe = 60 % and 2C-I = 28 % *vs* 25I-NBOMe = 70 %) [23].

**Table 1:** Binding affinity, activation potency, and activation efficacy of 2C and NBOMe drugs on 5-HT receptors (adapted from [25]).

**Drug /** **Receptor**	**5-HT**_**1A**_	**5-HT**_**2A**_	**5-HT**_**2B**_	**5-HT**_**2C**_	**Model**
	**Receptor Binding** K_i_ (nM)				
**2C-B**	240	8.6	-	47	HEK293 cells expressing human 5-HT_1A_, 5-HT_2A_ and 5-HT_2C_ receptors
**2C‐T‐7**	520	6.5	-	39	
**25B-NBOMe**	3600	0.5	-	6.2	
**25T7-NBOMe**	1800	1.1	-	6.4	
**LSD**	3	4.2	-	15	
	**Activation potency** EC_50_ (nM)				
**2C-B**	-	80	130	-	NIH-3T3 and HEK293 cells expressing human 5-HT_2A_ and 5-HT_2B_ receptors, respectively
**2C‐T‐7**	-	130	350	-	
**25B-NBOMe**	-	40	10	-	
**25T7-NBOMe**	-	260	310	-	
**LSD**	-	260	12,000	-	
	**Activation efficacy** % maximum				
**2C-B**	-	45	89	-	
**2C‐T‐7**	-	76	45	-	
**25B-NBOMe**	-	28	19	-	
**25T7-NBOMe**	-	41	14	-	
**LSD**	-	28	71	-

**Table 2:** Summary of drug action on 5-HT_2_ receptors.

**Drug /** **Receptor**	**5-HT**_**2A**_	**5-HT**_**2B**_	**5-HT**_**2C**_	**Model (Ref)**
**2C-B**	**partial agonist** **∼ 5–10 %** (weak response for PLC and PLA_2_ pathways)	-	**partial agonist** **∼ 40–50 %** (PLC and PLA_2_ pathways)	CHO cells expressing human 5-HT_2A_ or 5-HT_2C_ receptors [28]
**2C-N**	**partial agonist** **∼ 20 %** (PLA_2_ pathway)	-		
**2C-D** **2C-I**	**partial agonist** **∼ 15–30 %** (PLC and PLA_2_ pathways)	-		
**2C-C** **2C-D** **2C-E** **2C‐T‐2**	**full agonist** **∼ 65–125 %** (PLC and PLA_2_ pathways)	**-**	**full agonist** **∼ 80–100 %** (PLC pathway)	HEK cells expressing the corresponding human 5-HT receptors [29]
**2C-I**	**full agonist** **∼ 80 %** (PLC pathway) **antagonist** **∼ 30 %** (PLA_2_ pathway)			
**25D-NBOMe** **25E-NBOMe** **25H-NBOMe** **25N-NBOMe**	**full agonist** **∼ 85–95 %** (PLC pathway)	**partial agonist** **∼ 40–50 %** (PLC pathway)	**full agonist** **∼ 90–100 %** (PLC pathway)	HEK cells expressing the corresponding human 5-HT receptors [24]
**2C‐T‐4** **2C‐T‐7**	**partial agonist** **∼ 50–60 %**	**partial agonist** **∼ 45–75 %**	**-**	HEK 293 cells expressing the corresponding human 5-HT_2B_ receptors; mouse embryonic fibroblasts expressing the human 5-HT_2A_ receptor [27]
**2C-B, 2C-C,** **2C-D, 2C-E,** **2C-I, 2C-N,** **2C‐T‐2, 2C‐T‐4, 2C‐T‐7,** **25B-NBOMe,** **25C-NBOMe,** **25D-NBOMe,** **25E-NBOMe,** **25I-NBOMe,** **25N-NBOMe, 25T2-NBOMe, 25T4-NBOMe, 25T7-NBOMe**	**partial agonist** **∼ 40–90 %**	**partial agonist** **∼ 45–90 %**	**-**	NIH-3T3 expressing the human 5-HT_2A_ receptors; HEK293 cells expressing the human 5-HT_2B_ receptors [25]
**2C-C** **2C-I** **25C-NBOMe** **25I-NBOMe**	**full agonist** **∼ 90–110 %**	**-**	**partial agonist** **∼ 30–70 %**	HEK 293 cells expressing the corresponding human 5-HT_2A_ and 5-HT_2C_ receptors [23]

Furthermore, Luethi et al. explored 5-HT_1A_, 5-HT_2A_, 5-HT_2B_ or 5-HT_2C_ receptor binding affinities and/or activation potencies of a series of 2C-T-R drugs – 2C-T-4, 2C-T-7 and less reported 2C-T-1, 2C-T-3, 2C-T-16, 2C-T-19, 2C-T-21.5, 2C-T-22, 2C-T-25, 2C-T-27, 2C-T-28, 2C-T-30, 2C-T-31, and 2C-T-33 (Table S1) – in HEK 293 cells transfected with the appropriate receptor (5-HT_1A_, 5-HT_2A_, 5-HT_2B_ or 5-HT_2C_) and in mouse embryonic fibroblasts that express the human 5-HT_2A_ receptor. All the tested drugs bound to the 5-HT_1A_ (range of K_i_ 660–2368 nM), 5-HT_2A_ (range of K_i_ 1.6–54 nM) and 5-HT_2C_ receptors (range of K_i_ 40–347 nM), effectively activating 5-HT_2A_ receptor (activation potency: EC_50_ ranging from 1.2 to 53 nM) with an activation efficacy between 2.8 % and 75 %. Except for 2C-T-27 and 2C-T-33, all drugs activated 5-HT_2B_ receptor (range of EC_50_ 44–3309 nM) with an efficacy between 28 % and 75 %. Drugs presented 17- to 830-fold greater affinity for 5-HT_2A_ over 5-HT_1A_ receptors and 4- to 44-fold greater affinity for 5-HT_2A_ over 5-HT_2C_ receptors, as reported by [25]. Drugs containing a 4-benzylthio substituent (2C-T-27, 2C-T-31, and 2C-T-33) presented the highest 5-HT_2A_ affinity but the lowest 5-HT_2A_ activation potency, suggesting that phenethylamines with bulky lipophilic substituents might have 5-HT_2_ antagonistic effects. In addition, drugs containing fluorine (2C-T-21.5, 2C-T-22, 2C-T-28, 2C-T-30, and 2C-T-31) showed reduced receptor affinity for 5-HT. For example, the binding affinity of 2C-T-19 and 2C-T-7 for 5-HT_2A_ and 5-HT_2C_ was higher than that of their monofluorinated analogs, 2C-T-30 and 2C-T-28, respectively. However, 2C-T-19 demonstrated reduced activation potential for 5-HT_2A_ and 5-HT_2B_ compared to 2C-T-30, while 2C-T-7 showed increased activation potential for 5-HT_2A_ and 5-HT_2B_ compared to 2C-T-28 [27]. Thus, these findings suggest that an increased binding affinity for 5-HT receptors does not directly correlate with drug activation potency.

Accordingly, in Chinese hamster ovary (CHO) cells expressing 5-HT_2A_ or 5-HT_2C_ receptors, Moya et al. reported, that 2C-D and 2C-I acted as partial 5-HT_2A_ agonists, demonstrating similar efficacy across both signaling pathways tested (PLA_2_-arachidonic acid and PLC-inositol phosphate, Figs. 2), 2C-N was partial 5-HT_2A_ agonist eliciting only PLA_2_-arachidonic acid release, and 2C-B was partial 5-HT_2A_ agonist but with weak responses for the PLC and PLA_2_ pathways. Regarding 5-HT_2C_ receptors, 2C-D, 2C-I, 2C-N, and 2C-B acted as partial agonists, with no preference for either pathway (Table 2) [28]. Moreover, Eshleman et al. reported for 2C-C, 2C-D, 2C-E, 2C-I, and 2C-T-2 low affinity at 5-HT_1A_ and strong affinity at 5-HT_2A_ and 5-HT_2C_ receptors. In the inositol phosphate assay, all drugs showed full agonism activity at 5-HT_2A_ and 5-HT_2C_ receptors. However, in the arachidonic acid release assay, all phenethylamines were 5-HT_2A_ full agonists, except 2C-I, which showed antagonist activity (Table 2) [29]. 25D-NBOMe, 25E-NBOMe, 25H-NBOMe, and 25N-NBOMe (Table S2) showed limited potency and efficacy at the 5-HT_1A_ receptor but demonstrated strong affinity and full efficacy at 5-HT_2A_ and 5-HT_2C_ receptors. When compared to 5-HT_2A_ or 5-HT_2C_ receptors, the drugs presented lower binding affinity, potency, and efficacy at the 5-HT_2B_ receptor (Table 2) [24]. This indicates that the inclusion of the *N*-benzyl group to the phenethylamines increases the affinity for 5-HT_2A_ and 5-HT_2C_ receptors (*e.g.*, 2C-D 5-HT_2A_ K_i_ = 23.9 nM *vs* 25D-NBOMe 5-HT_2A_ K_i_ = 0.22 nM and 2C-D 5-HT_2C_ K_i_ = 12.7 nM *vs* 25D-NBOMe 5-HT_2C_ K_i_ = 0.69 nM) [24], [29].

However, in *Xenopus laevis* oocytes microinjected with 5-HT_2A_ or 5-HT_2C_ receptors rat clones, Villalobos et al. demonstrated that several 2C drugs function as strong and reversible antagonists of the 5-HT_2A_ receptor, with a potency ranking of 2C-I > 2C-B > 2C-D > 2C-H. No antagonism on 5-HT_2C_ receptors was observed [30].

Overall, 2C and NBOMe drugs are relatively selective for 5-HT_2_ receptors (Table 1), with most information pointing to a full agonist or partial agonist action on these receptors (Table 2). Variations in classification can result from the use of different concentrations and different models used to express 5-HT receptors, and the characterized second messenger systems (e.g., IP_3_ and DAG, Fig. 2).

#### 2.1.2 Effects on other transporters and receptors

Beyond interactions with 5-HT receptors, the impact of several drugs on MAO-A and MAO-B, monoamine transporters – serotonin (SERT), dopamine (DAT) and norepinephrine (NET), and monoamine receptors – adrenergic (α_1A_ and α_2A_), dopaminergic (D_1–3_), histaminergic (H_1_) and trace amine-associated receptor-1 (TAAR_1_) have also been investigated [24], [25], [27], [29], [31], [32], [33], [34].

Wagmann et al. evaluated, *in vitro* the potential for MAO-A and MAO-B inhibition of a set of 2C drugs – 2C-B, 2C-D, 2C-E, 2C-H, 2C-I, 2C-N, 2C-P, 2C-T-2, 2C-T-7 and 2C-T-21 using microsomes from insect cells infected with wild-type baculovirus (supersomes), as well as supersomes expressing human MAO-A or MAO-B. Screening results for MAO inhibition revealed that 2C-B, 2C-I and 2C-T-7 reduced MAO-A activity, with IC_50_ values between 46 and 125 µM), values much higher than the 0.2 µM IC_50_ observed for the known MAO-A inhibitor 5-IT. Furthermore, 2C-B, 2C-D, 2C-E, 2C-H, 2C-I, 2C-N, and 2C-T-7 reduced MAO-B activity, with IC_50_ values between 1.7 and 180 µM, whereas selegiline, a known MAO-B inhibitor, showed an IC_50_ value of 0.017 µM [31]. Moreover, a prior study found that MAO-A and MAO-B are involved in the deamination of 2C drugs, suggesting that these drugs may be vulnerable to cross-reactions with MAO inhibitors, potentially leading to increased plasma concentrations and a higher risk of adverse effects [35].

Many drugs can increase dopamine, norepinephrine, and 5-HT brain levels through the inhibition of monoamine uptake transporters (SERT, DAT and NET). Generally, 2C and NBOMe drugs present higher binding affinity for SERT than for DAT or NET, with DAT consistently being the transporter with lower binding affinity. Moreover, relative to 2C drugs, NBOMe drugs present higher binding affinity for all the transports. However, this affinity is still low, as observed in Table 3. Comparable findings were obtained for the inhibition potency, with the lower and higher IC_50_ values being observed for SERT and DAT, respectively. However, when compared to known monoamine transporter inhibitors – methylphenidate (DAT), reboxetine (NET) and citalopram (SERT) – these values are dismissible (Table 3) [24], [25], [27], [29], [34]. Eshleman et al. also evaluated drug induced release of preloaded [^3^H]neurotransmitter from human DAT, SERT and NET, showing small to none releasing efficacy for both 2C and NBOMe drugs (% maximum release below 25 for all the drugs) [24], [29]. Thus, based on these data, it seems unlikely that these drugs exert their psychoactive effects through substantial modulation of monoamine uptake or release.

**Table 3:** Examples of monoamine transporter affinity and inhibition for 2C and NBOMe drugs.

**Drug /** **Transporter**	**DAT**	**NET**	**SERT**	**Model**	**Ref**
	**Transporter Binding** K_i_ (nM)				
**2C-D**	> 10,000	> 8500	> 10,000	HEK293 cells expressing human DAT, NET or SERT	[29]
**2C-E**	> 10,000	> 7600	> 10,000		
**2C-I**	> 1,0000	> 10,000	950		
**25D-NBOMe**	34,500	6700	1780		[24]
**25E-NBOMe**	19,600	5400	1590		
**2C-B**	> 30,000	31,000	9700		[25]
**25B-NBOMe**	7200	1100	840		
**2C-I**	> 30,000	15,000	4900		
**25I-NBOMe**	5400	1300	1000		
**2C‐T‐4**	> 8710	> 9710	> 8580		[27]
**2C‐T‐7**	> 8710	> 9710	> 8580		
	**Inhibition potency** IC_50_ (nM)				
**2C-D**	> 10,000	> 9200	> 8400	HEK293 cells expressing human DAT, NET or SERT	[29]
**2C-E**	> 10,000	> 9800	> 7500		
**2C-I**	> 9600	> 8200	5600		
**25D-NBOMe**	> 85,000	1170	1024		[24]
**25E-NBOMe**	34,000	2310	1440		
**2C-B**	231,000	44,000	18,000		[25]
**25B-NBOMe**	117,000	6700	7100		
**2C-I**	126,000	22,000	13,000		
**25I-NBOMe**	65,000	10,000	6800		
**Methylphenidate**	120	-	-		
**Reboxetine**	-	36	-		
**Citalopram**	-	-	45		
**2C-B**	132,000	122,000	4700		[34]
**25B-NBOMe**	99,000	11,000	4900		
**25I-NBOMe**	53,000	11,000	4000		
**Cocaine**	1000	1100	1400		
**Methoxetamine**	24,000	15,000	2400		

Among the monoamine receptors, 2C and NBOMe substances displayed higher binding affinity for adrenergic α_2A_ and TAAR_1rat_ receptors (overall K_i_ < 1000 nM). Compared to a set of 2C drugs (2C-B, 2C-C, 2C-D, 2C-E, 2C-H, 2C-I, 2C-N, 2C-P, 2C-T-2, 2C-T-4, 2C-T-7), NBOMe analogs exhibit higher binding affinities for adrenergic α_1A_ receptors (K_i_ < 1000 nM for NBOMe *vs* K_i_ > 3500 nM for 2C drugs), dopaminergic D_1‐3_ receptors (with low affinity for both 2C and NBOMe drugs), and histaminergic H_1_ receptors (overall high NBOMe affinity) [25]. Accordingly, Luethi et al. reported low affinity for adrenergic α_1A_ receptors (K_i_ > 2297 nM) and dopaminergic D_2_ receptors (K_i_ > 4400 nM) for a series of 2C-T-R drugs. All phenethylamines bound to rat (range of K_i_ 4.8–68 nM) and mouse (range of K_i_ 55–2337 nM) TAAR_1_, but none of them activated the human TAAR_1_ (EC_50_ > 30,000 nM) [27]. In line with these results, 2C-C, 2C-D, 2C-E, 2C-I, and 2C-T-2 exhibited no quantifiable affinity for dopamine D_1_-D_3_ receptors (cell lines expressing these human receptors), suggesting that their psychoactive effects are not driven by direct dopamine receptor activity [29].

Interestingly, Åstrand et al. and Deventer et al. identified a μ-opioid receptor activity for several 2C and NBOMe drugs, including 2C-I, 25I-NBOMe (and its metabolite – 2-desmethyl-25I-NBOMe), 25B-NBOMe, 25C-NBOMe, 25D-NBOMe and 25E-NBOMe (25 µM). Moreover, Åstrand et al. reported that 25I-NBOMe (7.5 µg/mL) also targets the cannabinoid receptor 1 (CB_1_, between the limit of detection and 50 % of full agonist signal). This indicates that phenethylamines can have off-target effects that must be considered when interpreting their adverse effects [36], [37].

Overall, the interactions with MAO and monoamine receptors and transporters reported for 2C and NBOMe drugs are weak and appear to have limited pharmacologically and toxicologically significance when compared to the interactions with serotoninergic receptors.

### 2.2 In vivo studies

#### 2.2.1 5-HT_2A_-dependent behaviors

Aligned with the *in vitro* findings, these drugs prompt 5-HT_2A_-mediated responses *in vivo*, including wet dog shakes (WDS), back muscle contractions (BMC), and head twitch responses (HTR) in rats and mice (Table 4) [9], [15], [23], [38], [39], [40], [41], [42], [43], [44].

**Table 4:** Summary of in vivo 5-HT_2A_-dependent behaviors.

**Movement**	**Drug**	**ED**_**50**_ (mg/kg)	**Doses** (mg/kg)	**Model and route of** **administration**	**Ref**
WDS	2C-C	0.173	**0.3**, 1, 3	Sprague-Dawley rats SC	[23]
	2C-I	0.690	1, **3**		
	25C-NBOMe	0.010	**0.03**, 0.1		
	25I-NBOMe	0.062	**0.1**, 0.3		
	25I-NBOMe	-	**1**, 3	Wistar-Han rats SC	[43]
BMC	2C-C	0.550	0.3, 1, **3**	Sprague-Dawley rats SC	[23]
	2C-I	0.192	0.3, **1**, 3		
	25C-NBOMe	0.021	0.03, **0.1**, 0.3		
	25I-NBOMe	0.037	0.03, 0.1, **0.3**		
HTR	2C-T−7	-	**1**, 3	NIH Swiss mice IP	[38]
	2C-B	0.72	1, **3**, 10	C57BL/6 J mice IP	[40]
	2C-I	0.83	1, **3**, 10	C57BL/6 J mice SC	[41]
	25I-NBOMe	0.078	0.1, 0.3, **1**		
	25I-NBOMe	-	0.3, **1**, 3, 10	Wistar-Han rats SC	[42]
	25H-NBOMe	-	0.3, 1, **3**	Wistar rats IP	[39]
	25B-NBOMe	-	1, **3**, 10	C57BL/6 J mice IP	[9]
	25E-NBOMe	-	0.1, 0.3, **1**, 3	C57BL/6 J mice IP	[15]

Bold highlights the most potent dose inducing the behaviors. IP (Intraperitoneal); SC (Subcutaneous).

Elmore et al. observed that 25C-NBOMe and 25I-NBOMe (0.01–0.3 mg/kg, SC) displayed 5- to 27-fold greater potency than their 2C analogs (2C-C and 2C-I, 0.1–3.0 mg/kg, SC) in eliciting WDS and BMC in rats (Table 4). Notably, a reverse U-shaped dose-response curve for WDS suggested that at higher doses, activation of other receptors (*e.g*., 5-HT_2C_) might counteract this behavior, as previously proposed [45]. Furthermore, pre-administration of M100907 (0.1 mg/kg, SC), a selective 5-HT_2A_ antagonist, eliminated both WDS and BMC, indicating that the observed behavioral effects are dependent on 5-HT_2A_ receptor activation [23]. Accordingly, Herian et al. showed that 25I-NBOMe (1 and 3 mg/kg, SC) induced WDS (Table 4). This effect was suppressed by 5-HT_2A_ and 5-HT_2C_ antagonists (M100907 and SB242084, 100 nM), but not by 5-HT_1A_ antagonist (WAY100635, 100 nM), suggesting the involvement of 5-HT_2A_ and 5-HT_2C_ receptors in 25I-NBOMe-elicited WDS [43].

5-HT_2A_ receptors activation by 2C and NBOMe drugs has been extensively explored in mice and rats using the HTR behavior, where the activation of this receptor causes a high-frequency paroxysmal head rotation related to hallucinogen behavior [46]. Fantegrossi et al. and Halberstadt and Geyer et al. showed that 2C-T-7 (0.3–1 mg/kg, IP), 2C-I (1–10 mg/kg, SC) and 25I-NBOMe (0.1–1 mg/kg, SC) significantly provoked HTR in mice (Table 4), with M100907 successfully blocking this response [38], [41].

Moreover, several research groups reported that 2C-B (0.3–10 mg/kg, IP), 25B-NBOMe (0.3–10 mg/kg, IP), 25E-NBOMe (0.1–3 mg/kg, IP), 25H-NBOMe (0.3–3 mg/kg, IP) and 25I-NBOMe (0.3–10 mg/kg, SC) induced HTR in rats (25I-NBOMe and 25H-NBOMe) and mice (2C-B, 25B-NBOMe and 25E-NBOMe), with the 1 mg/kg 25I-NBOMe and 25E-NBOMe, and the 3 mg/kg 2C-B, 25B-NBOMe and 25H-NBOMe doses showing the most potent HTR induction (Table 4) [9], [15], [39], [40], [42]. Interestingly, Marcher-Rørsted et al. demonstrated that removing the 2- or 5-methoxy groups in 2C-B significantly reduced HTR potency in C57BL/6 J mice, suggesting the 2,5-dimethoxyphenethylamine motif is crucial for 5-HT_2A_ receptor activation. Despite this decrease in HTR potency, only a slight decrease was noted in the binding affinities for the 5-HT_2A_, 5-HT_2B_ and 5-HT_2C_, as well as in functional potency at the 5-HT_2A_ and 5-HT_2C_ receptors [47].

In contrast, Moya et al. stated that 2C-I (∼ 2.6 mg/kg,IP), 2C-B (∼ 2.2 mg/kg,IP), and 2C-D (∼1.6 mg/kg,IP) (equimolar doses of 8.4 µmol/kg) failed to induce HTR in Sprague-Dawley rats (even with doses of 15 mg/kg, IP), establishing no correlation between 5-HT_2A_ partial agonist behavior (formerly mentioned) and the induction of HTR [28].

Overall, a higher but dose-independent number of WDS, BMC, and HTR was perceived following the administration of several 2C and NBOMe drugs (Table 4). In some cases, this effect was blocked by a 5-HT_2A_ receptor antagonist, clarifying this receptor impact in triggering these behavioral responses.

Recently, Halberstadt et al. proposed that the hallucinogen potency in HTR assays (performed in C57BL/6 J mice) is strongly interrelated to their effects in rats and humans, suggesting that HTR studies in mice can help explore the pharmacological/toxicological interactions behind the hallucinogenic effects of drugs in humans [48]. Indeed, the same group investigated the SAR relationship of a set of 2C drugs using HTR dose-response studies in C57BL/6 J mice. They concluded that 2C-T (2,5-dimethoxy-4-methylthiophenethylamine, Table S1) HTR potency increased with the insertion of an α-methyl group (ALEPH: 2,5-dimethoxy-4-methylthioamphetamine) and by extending the 4-methylthio group (2C-T-2 and 2C-T-7). However, fluorination of the 4-position alkylthio chain (2C-T-21 and 2C-T-28, Table S1) and the existence of a 4-allylthio group (2C-T-16, Table S1) decreased HTR potency. Moreover, as formerly reported [27], bulky 4-substituents such as 4-benzylthio analogs (2C-T-27 and 2C-T-33, Table S1) had little effect in the HTR paradigm [49].

#### 2.2.2 Additional behavioral responses and drug discrimination studies

The capacity of psychedelic phenethylamines to change locomotor activity has been assessed in rats and mice across various studies [11], [12], [13], [15], [29], [39], [50], [51]. Drug discrimination techniques are frequently used for assessing drug abuse liability, since they correlate with the possibility of human use [29], [38], [50], [52].

Eshleman et al. assessed the impact of six substituted phenethylamines: 2C-C, 2C-D, 2C-E, 2C-I, and 2C-T-2 on mouse locomotor activity (Table 5). Following IP injection, 2C-C (30 and 100 mg/kg), 2C-I (3, 10, and 30 mg/kg) and 2C-T-2 (3 and 10 mg/kg) produced a dose- and time-dependent reduction in mice activity. However, 2C-D and 2C-E showed a biphasic response: high doses (10 and 30 mg/kg) were depressant, while low doses (3 mg/kg for 2C-D, 0.3 and 1 mg/kg for 2C-E) were stimulant [29]. Similarly, Kim et al. observed reduced locomotor activity in mice administered with 2C-C and 2C-P (30 mg/kg, IP) (Table 5) [12]. Beyond locomotor effects, Eshleman et al. evaluated drug discrimination, observing that, with the exception of 2C-T-2 (which only partially substituted for DMT), all tested phenethylamines totally substituted for the discriminative effects of at least one training drug, including DOM, DMT, LSD, or MDMA (Table 6). However, none substituted for (+)-methamphetamine, indicating low psychostimulant potential [29]. Moreover, Fantegrossi et al. reported that 2C-T-7 (1 and 3 mg/kg, IP) elicited up to 75 % LSD-appropriate responses in rats trained to distinguish LSD (Table 6). In rats trained to recognize 2C-T-7 (1 mg/kg, IP), the drug (0.1, 0.3 and 1 mg/kg, IP) induced a dose-dependent 2C-T-7-appropriate responding, and M100907 (0.05 mg/kg, IP) pretreatment successfully blocked the discriminative effects produced by 2C-T-7 (1 mg/kg, IP) [38].

**Table 5:** Summary of locomotor activity alterations.

**Drug**	**Increased locomotor activity** dose (mg/kg)	**Decreased locomotor activity** dose (mg/kg)	**Model and route of** **administration**	**Ref**
2C-C	-	30, **100**	Swiss-Webster mice IP	[29]
2C-D	**3**	10, **30**		
2C-E	0.3, **1**	10, **30**		
2C-I	-	3, 10, **30**		
2C-T−2	-	3, **10**		
2C-C	-	**30**	CD-1 mice IP	[12]
2C-P	-	**30**		
25B-NBOMe	-	2.5, 5, **10**	Swiss-Webster mice IP	[50]
25C-NBOMe	-	0.5, 1, 2.5, **5**		
25I-NBOMe	-	2.5, 5, 10, **25**		
MDMA	5, 10, 25, **50**	-		
25C-NBOMe	0.03, **0.1**, 0.3	-	Sprague-Dawley rats IP	[55]
25I-NBOMe	0.001, 0.01, **0.1**, 1	**10**	CD-1 mice IP	[53]
LSD	0.001, 0.01, 0.1, **1**	**10**		
25H-NBOMe	**0.1**	-	C57BL/J6 mice IP	[13]
Meth	**1**	-		
25N-NBOMe	-	**3**	Wistar rats IP	[39]

Bold highlights the most potent dose inducing locomotor activity alterations. IP (Intraperitoneal).

**Table 6:** Summary of discriminative stimulus effects.

**Training Drug**	**Drugs that totally substituted the training drug** ≥ 80 % of drug appropriate responding	**Model and route of** **administration**	**Ref**
LSD (0.1 mg/kg)	2.5 mg/kg of 2C-E: 97 % 2.5 mg/kg of 2C-D: 83 % 5 mg/kg of 2C-I: 80 %	Sprague-Dawley rats IP	[29]
DMT (5 mg/kg)	2.5 mg/kg of 2C-D: 100 % 2.5 mg/kg of 2C-I: 93 % 5 mg/kg of 2C-E: 80 %		
DOM (0.5 mg/kg)	2.5 mg/kg of 2C-D: 100 % 2.5 mg/kg of 2C-E: 97 % 2.5 mg/kg of 2C-C: 82 %		
MDMA (1.5 mg/kg)	10 mg/kg of 2C-E: 100 % 2.5 mg/kg of 2C-C: 89 %		
DOM (0.5 mg/kg)	0.5 mg/kg of 25B-NBOMe: 83 % 1 mg/kg of 25C-NBOMe: 97 %	Sprague-Dawley rats IP	[50]
MDMA (1.5 mg/kg)	0.5 mg/kg of 25B-NBOMe: 92 %		
DOM (0.32 mg/kg)	0.32 mg/kg of 2C-T−7: > 90 %	Rhesus Monkeys SC	[52]
**Training Drug**	**Drugs that partially substituted the training drug** ≥ 40 % of drug appropriate responding	**Model and route of administration**	**Ref**
LSD (0.1 mg/kg)	5 mg/kg of 2C-C: 75 %	Sprague-Dawley rats IP	[29]
DMT (5 mg/kg)	5 mg/kg of 2C-C: 75 % 2.5 mg/kg of 2C-T−2: 73 %		
MDMA (1.5 mg/kg)	2.5 mg/kg of 2C-D: 61 % 2.5 mg/kg of 2C-I: 65 %		
DOM (0.5 mg/kg)	1 mg/kg of 25B-NBOMe: ∼ 50 % 2.5 mg/kg of 25I-NBOMe: 74 %	Sprague-Dawley rats IP	[50]
MDMA (1.5 mg/kg)	1 mg/kg of 25B-NBOMe: ∼ 50 % 1 mg/kg of 25C-NBOMe: 67 % 1 mg/kg of 25I-NBOMe: 78 %		
LSD (0.1 mg/kg)	1 and 3 mg/kg of 2C-T−7: 75 %	Fischer-344 rats IP	[38]

LSD (lysergic acid diethylamide), DMT (N,N,-dimethyltryptamine); DOM (dimethyltryptamine); MDMA (3,4-methyl enedioxy methamphetamine); SC (Subcutaneous); IP (Intraperitoneal).

Research has shown varied responses in locomotor activity among NBOMe compounds. Jo et al. found that 25H-NBOMe (0.1 mg/kg, IP) augmented activity in mice, peaking at 0.1 mg/kg before declining at higher doses (0.5–5 mg/kg, IP) (Table 5), consistent with a typical bell-shaped response curve [13]. Ferry et al. found that 25N-NBOMe only reduced locomotion at the highest tested dose (3 mg/kg, IP) in rats (Table 5), while Kim et al. and Seo et al. observed no significant effects on mice administred 25E-NBOMe (0.1–3 mg/kg, IP) or 25N-NBOMe (0.3–3 mg/kg, IP) [11], [15]. Gatch et al. found that 25B-NBOMe (2.5–10 mg/kg, IP), 25C-NBOMe (0.5–5 mg/kg, IP), and 25I-NBOMe (2.5–25 mg/kg, IP) induced a decrease in mice locomotor activity that was both time- and dose-dependent (Table 5). Furthermore, 25B-NBOMe (0.5 mg/kg) and 25C-NBOMe (1 mg/kg) achieved complete substitution for DOM, while only 25B-NBOMe (0.5 mg/kg) fully substituted for MDMA, suggesting that 25B-NBOMe, DOM, and MDMA have overlapping toxicological profiles (Table 6). The inability of 25I-NBOMe to completely substitute for DOM or MDMA may be attributed to its depressive effect rather than insufficient discriminatory ability. Thus, all drugs are more likely to be used recreationally as hallucinogens rather than as MDMA alternatives, though 25B-NBOMe shows potential for both hallucinogenic and entactogenic use. However, the inverted U-shaped dose-response curve of 25B-NBOMe may reduce its popularity due to the limited window for desirable effects [50].

Interestingly, chronic administration of 25I-NBOMe (0.3 mg/kg, SC, for 7 consecutive days) decreased locomotor activity and exploration actions of Wistar-Han rats, relative to both control and single-dose (0.3 mg/kg, SC) groups. These rats also showed memory deficits in the novel object recognition test, with single-dose animals spending less time with the novel object, while those receiving repeated doses explored both the familiar and novel objects equally. Both single and repeated doses increased the time spent in the dark zone during the light-dark box test, suggesting anxiety-like behavior [51].

Further studies explored sensorimotor and vocal responses. Tirri et al. observed that in CD-1 mice, 2C-H, 2C-B, 2C-I, and their NBOMe analogs (0.001–10 mg/kg, IP) reduced visual and acoustic responses, decreasing the startle reflex (only NBOMe drugs), and increasing reaction time. Only 25I-NBOMe considerably changed the spontaneous locomotor activity, where lower and intermediate doses (0.001–1 mg/kg) increased this behavior, and higher doses (10 mg/kg) reduced it (Table 5). In general, 25I-NBOMe and 25B-NBOMe produced stronger responses compared to 25H-NBOMe and 2C drugs [53]. Complementing the findings on 25I-NBOMe, Miliano et al. reported sex-dependent sensorimotor differences in Sprague-Dawley rats (0.1–1 mg/kg, IP). Both sexes experienced reduced visual responses, while only male rats showed a decrease in acoustic and tactile responses. At the highest dose, both showed a significant increase in pain threshold to mechanical stimulus (tail pinch test), with the response being more pronounced in males, and body core temperature was only increased in females. Additionally, in both sexes the drug inhibited prepulse inhibition (PPI) and impaired startle amplitude [54]. Jeon et al. quantified the ultrasonic vocalizations of C57BL/6 J mice following 25I-NBOMe (0.3 mg/kg, IP) administration, showing higher frequencies than the vehicle animals [10]. Similarly, Sprague-Dawley rats treated with 25C-NBOMe significantly reduced PPI (0.1 mg/kg, IP) without disturbing startle response, along with a dose-dependent, reversed U-shaped increase in locomotion (0.03, 0.1, and 0.3 mg/kg, IP) (Table 5) [55]. Custodio et al. explored the influence of 25B-NBOMe (1 mg/kg) on brain wave activity in C57BL/6 J mice using electroencephalography. They observed an increase in delta waves and a reduction in gamma wave activity, effects that were normalized by dopamine receptor antagonists, indicating a potential role of the dopamine system in mediating these changes [9].

Although the best *in vivo* model to study psychedelics actions in humans are the non-human primates, almost no information has been published. Regarding 2C drugs, only one relevant study was found in the literature. Li et al. investigated, in rhesus monkeys, the discriminative stimulus (trained animals to distinguish DOM from vehicle) effects of 2C-T-7 administrated alone or paired with three 5-HT antagonists: MDL100907 and ketanserin (both with a higher affinity for 5-HT_2A_ than 5-HT_2C_ receptors), and ritanserin (similar affinity for 5-HT_2A_ and 5-HT_2C_ receptors). The administration of 2C-T-7 caused an incremental increase in DOM-associated lever presses. Despite the different 5-HT_2_ binding selectivity, all three antagonists blocked the discriminative stimulus effect of the drug, with potency correlating to 5-HT_2A_ receptor affinity, leading the authors to suggest that 2C-T-7 discriminative effects in rhesus monkeys are largely, if not fully, mediated by 5-HT_2A_ receptors [52], as previously observed for other *in vivo* models [38].

#### 2.2.3 Development of tolerance and cross-tolerance

Knowing that frequent administration of psychedelics causes a fast development of tolerance [56], [57] and cross-tolerance [58], [59] phenomena, Smith et al. investigated these effects in NIH Swiss mice by measuring the drug-induced HTR with two phenethylamines: DOI (2,5-dimethoxy-4-iodoamphetamine) and 2C-T-7. After administering DOI or 2C-T-7 at 1.0 mg/kg IP for five consecutive days, mice developed gradual HTR tolerance. Furthermore, cross-tolerance was evident in DOI-tolerant mice treated with 1.0 mg/kg (IP) for three days, then tested with various 2C-T-7 doses (0.3–10 mg/kg, IP) [44]. More recently, Herian et al. reported that chronic 25I-NBOMe administration (0.3 mg/kg, SC for 7 days) caused a gradual reduction of the WDS response over time, signaling the development of tolerance within the first days of treatment [51].

## 3 Abuse potential

A question that is not yet clarified is whether these drugs have abuse potential. Recent studies in rodents evaluated the rewarding and reinforcing effects of 2C-C, 2C-P, 25B-NBOMe, 25D-NBOMe, 25E-NBOMe, 25H-NBOMe, 25I-NBOMe and 25N-NBOMe, using two behavioral tests: conditioned place preference (CPP) and self-administration (SA) paradigms (Table 7). In SA, two parameters were evaluated: the total of infusions and frequency of active (rewarded) *versus* inactive (non-rewarded) lever presses per session.

**Table 7:** Rewarding and reinforcing effects of 2C and NBOMe drugs.

**Drug**	**CPP**	**SA** dose (mg/kg)			**Model**	**Ref**
	C57BL/6 J mice dose (mg/kg, IP)	↑ number of infusions	active lever presses	inactive lever presses	IV	
2C-C	10	0.03	-	0.03	Sprague Dawley rats	[12]
2C-P	10	0.01, 0.03	0.01, 0.03	0.01	Sprague Dawley rats	[12]
25B-NBOMe	1	0.03, 0.1, 0.3	0.03, 0.1, 0.3	-	Sprague Dawley rats	[9]
25D-NBOMe	1	0.03	0.03	-	Sprague Dawley rats	[14]
25E-NBOMe	0.1	0.03	-	0.01	Sprague Dawley rats	[15]
25H-NBOMe	0.05, 0.1, 0.5	0.01	0.01	-	C57BL/6 J mice	[13]
25I-NBOMe	0.3	-	-	-	Sprague Dawley rats	[10]
25N-NBOMe	3	0.01	-	-	Sprague Dawley rats	[11]

CPP (conditioned place preference); IP (Intraperitoneal); IV (Intravenously); SA (self-administration).

IP administration of 2C-C (10 mg/kg), 2C-P (10 mg/kg), 25B-NBOMe (1 mg/kg), 25D-NBOMe (1 mg/kg), 25E-NBOMe (0.1 mg/kg), 25H-NBOMe (0.05, 0.1 and 0.5 mg/kg), 25I-NBOMe (0.3 mg/kg) and 25N-NBOMe (3 mg/kg) induced CPP in C57BL/6 J mice (Table 7), with effects comparable in intensity to the control - methamphetamine (1 mg/kg), suggesting that these drugs exert a rewarding effect [9], [10], [11], [12], [13], [14], [15].

The observed CPP effect induced by 25B-NBOMe was blocked by dopamine D_1_ and D_2_ receptor (DRD_1_ and DRD_2_) antagonists, but not by a 5-HT_2A_ receptor antagonist, suggesting that the dopamine system may play a part in the rewarding effects of 25B-NBOMe [9]. Kim et al. also found that pretreatment with a DRD_1_ antagonist constrained the place preference induced by 25E-NBOMe, whereas pretreatment with a DRD_2_ antagonist did not affect the drug's rewarding effects [15].

Concerning the SA tests, when compared to control animals, 2C-C (0.03 mg/kg/infusion), 25E-NBOMe (0.03 mg/kg, IV), and 25N-NBOMe (0.01 mg/kg, IV) significantly augmented the total number of infusions, but did not affect the number of active lever presses (Table 7). 2C-P (0.01 and 0.03 mg/kg, IV), 25B-NBOMe (0.03, 0.1, and 0.3 mg/kg, IV), 25D-NBOMe (0.03 mg/kg, IV), and 25H-NBOMe (0.01 mg/kg, IV) significantly raised both parameters, while 25I-NBOMe (0.03 mg/kg, IV) had no significant effect on either parameter (Table 7). Only 2C-C (0.03 mg/kg, IV), 2C-P (0.01 mg/kg, IV), and 25E-NBOMe (0.01 mg/kg, IV) significantly increased inactive lever pressing (Table 7). However, these inactive lever presses occurred only during the early sessions and decreased as the sessions progressed [9], [10], [11], [12], [13]. Altogether, the higher number of infusions and active lever presses suggest that these drugs have reinforcing properties.

The reinforcing effects of addictive drugs are primarily mediated through the dopamine system, with these drugs increasing dopamine levels via direct or indirect actions on dopamine neurons in the ventral tegmental area (VTA). This increase leads to dopamine release in the nucleus accumbens (NAc), a key brain region involved in reward signaling [60].

Accordingly, 2C-C and 2C-P administration to C57BL/6 J mice (10 mg/kg, IP) significantly decreased DRD_2_ expression in the NAc (without changes in DRD_1_) and DRD_1_ expression in the medial prefrontal cortex (without changes in DRD_2_ expression). Both drugs significantly decreased the expression of DAT and augmented phosphorylated-DAT (*p*-DAT, increase *p*-DAT levels leads to increased dopamine reuptake) levels and c-Fos (marker of neuronal activity associated with drug addiction) induction in the NAc. Moreover, 2C-P significantly increased *p*-DAT levels in the medial prefrontal cortex [12].

Changes in dopamine levels has been noted for 25B-NBOMe, 25D-NBOMe, 25E-NBOMe, 25H-NBOMe and 25I-NBOMe in the NAc and striatal regions of rodents (Table 8) [9], [10], [13], [14], [15]. For instance, Jo et al. reported an increase in extracellular dopamine levels in the striatum of Sprague-Dawley rats after IP administration of 10 mg/kg 25H-NBOMe, lasting up to 60 minutes [13]. Using the same model, Lee et al. observed that 25D-NBOMe (1, 3 and 10 mg/kg, IP) elevated the extracellular dopamine levels in the NAc, along with elevated levels of its metabolites, DOPAC and HVA. Moreover, 25D-NBOMe (1 mg/kg) decreased DRD_2_ and DAT expression, and increased the DRD_1_, tyrosine hydroxylase (TH, key enzyme in dopamine synthesis and important in controlling its levels), and *p*-DAT expression levels in the NAc of C57BL/6 J mice [14].

**Table 8:** Summary of dopamine signaling pathways.

**Drug**	**Brain regions**	**DA levels**	**DRD**_**1**_ **levels**	**DRD**_**2**_ **levels**	**DAT** **levels**	***p*****-DAT** **levels**	**TH** **levels**	**Ref**
2C-C	NAc	-	NA	↓	↓	↑	-	[12]
	mPFC	-	↓	NA	NA	↑	-	
	Striatum	-	-	-	-	-	NA	
2C-P	NAc	-	NA	↓	↓	↑	-	
	mPFC	-	↓	NA	NA	NA	-	
	Striatum	-	-	-	-	-	NA	
25B-NBOMe	NAc	↑	↑	↓	-	-	-	[9]
	VTA	-	-	↓	↓	-	-	
25D-NBOMe	NAc	↑	↑	↓	↓	↑	↑	[14]
25E-NBOMe	NAc	↓	↑	NA	↑	-	-	[15]
25H-NBOMe	Striatum	↑	-	-	-	-	-	[13]
25I-NBOMe	Striatum	↑	-	-	-	-	-	[10]
	Frontal cortex	↑	-	-	-	-	-	[42]
25N-NBOMe	NAc	-	NA	↓	↓	↑	↓	[11]
	Striatum	-	NA	↓	NA	↑	NA	

NA (not altered); NAc (nucleus accumbens); mPFC (medial prefrontal cortex); VTA (ventral tegmental area); DA (dopamine); DRD_1_ (dopamine D1 receptor); DRD_2_ (dopamine D2 receptor)); DAT (dopamine transporter); *p*-DAT (phosphorylated-DAT); TH (tyrosine hydroxylase).

Repeated administration of C57BL/6J mice with 25N-NBOMe (3 mg/kg) decreased DRD_2_, DAT, and TH expression in the NAc, while increasing *p*-DAT with no change in DRD_1_ levels. In the dorsal striatum, 25N-NBOMe reduced DRD_2_ and increased *p*-DAT levels, but did not affect DRD_1_, DAT, or TH expression [11]. Furthermore, dopamine levels were also increased in the NAc tissue of C57BL/6 J mice administered with 25B-NBOMe (1 mg/kg for 7 days). In this region, 25B-NBOMe significantly increased DRD_1_ and decreased DRD_2_ expression, and in the VTA, it significantly decreased DAT and DRD_2_ without affecting TH expression. This drug also modified the expression of transcription factors associated with neuroadaptation, increasing ΔFosB and phosphorylated cAMP-responsive element-binding protein (*p*-CREB) and decreasing brain-derived neurotrophic factor (BDNF) in the NAc [9]. On the other hand, Kim et al. found that 25E-NBOMe administration (0.1 mg/kg IP in tissue and a series of progressively higher doses for extracellular levels) decreased dopamine in the NAc of C57BL/6 J mice, while DOPAC and HVA remained unaltered. In the NAc, 25E-NBOMe (0.1 mg/kg IP) significantly increased DAT and DRD_1_ but not DRD_2_ levels and elevated dopamine-related signaling proteins DARPP32 and *p*-CREB [15].

Exposure of C57BL/6 J mouse striatal synaptosomes to 25I-NBOMe (0.1, 10, and 100 μM) significantly enhanced dopamine levels. Administration of 25I-NBOMe (0.3 mg/kg) also increased expression of serum- and glucocorticoid-regulated kinase 1 (SGK_1_) and decreased period circadian protein homolog 2 (PER_2_), both potential biomarkers for addiction-related behaviors [10]. In addition, 25I-NBOMe (0.3–10 mg/kg, SC) increased extracellular dopamine and 5-HT levels in the frontal cortex of Wistar-Han rats, with the strongest effect observed at 3 mg/kg. It also elevated brain tissue levels of 5-HT and its metabolite, 5-HIAA, without affecting tissue levels of dopamine or its metabolites (DOPAC and HVA). Moreover, 25I-NBOMe (1, 3, and 10 mg/kg, SC) increased extracellular glutamate levels in the rat frontal cortex [42] – a mechanism characteristic of many psychedelic drugs, often associated with 5-HT_2A_ receptor activation and thought to stimulate glutamate-dependent pyramidal neuron activity in the prefrontal cortex [61]. In a follow up study, Herian et al. showed that the increases in dopamine, 5-HT and glutamate triggered by 25I-NBOMe (1 and 3 mg/kg, IP) were diminished by 5-HT_2A_ and 5-HT_2C_ receptor antagonists (M100907 and SB242084, respectively). However, 5-HT_1A_ receptor antagonist WAY100635 only inhibited dopamine and 5-HT release. This suggests that 5-HT_2A_ and 5-HT_2C_ receptors play a primary role in 25I-NBOMe-induced neurotransmitter release [43]. Later, the same group studied the impact of repeated administration of 25I-NBOMe (0.3 mg/kg, SC for 7 days) on dopamine, 5-HT, glutamate and acetylcholine release across three brain regions. In frontal cortex, rats repeatedly exposed to 25I-NBOMe showed a weaker dopamine, 5-HT, and glutamate response to a challenge dose of the drug (0.3 mg/kg), compared to those given a single dose. In contrast, in the NAc, repeated exposure led to a stronger response across dopamine, 5-HT, and glutamate systems than in single-dosed animals. In the striatum, repeated drug administration heightened dopamine and 5-HT responses to the challenge dose relative to acute treatment, while reducing the glutamatergic response. For acetylcholine, extracellular levels increased in response to the challenge dose in all three regions among animals repeatedly treated with 25I-NBOMe compared to the acutely treated group [51].

Interestingly, Miliano et al. provided the firs report on sex-dependent differences in the neurochemical profile of 25I-NBOMe in Sprague-Dawley rats (0.1–1.0 mg/kg, IP), identifying distinct sex-specific patterns in dopamine and 5-HT transmission within the NAc shell and core and in the medial prefrontal cortex. In male rats, dopamine transmission was affected in the NAc shell and core at the lowest dose (0.3 mg/kg, IP), with no alterations observed in the medial prefrontal cortex. For 5-HT transmission, no changes were perceived in any of the tested areas. In female rats, dopamine transmission was affected in the NAc shell and in the medial prefrontal cortex at the lowest dose, while no effects were observed in the NAc core. As in males, no effect on 5-HT transmission was observed in any of the tested areas, although there was a tendency toward increased levels in the NAc shell [54]. In line with previously reported studies, the increase in dopamine transmission, especially in the NAc (Table 8) suggests an abuse potential for these drugs.

Noteworthy, while there are no specific studies correlating 5-HT_2C_ receptor activation with the prevention of the abuse potential of 2C and NBOMe drugs, this possibility cannot be excluded, as similar effects have been observed for other psychedelic drugs. 5-HT_2C_ receptors are involved in the regulation of reward processing and can moderate the rewarding effects of drugs. For example, the use of 5-HT_2C_ receptor agonists has been found to decrease substance use actions, including the SA of ethanol, cocaine, and nicotine in rodent models [62]. As previously mentioned, Herian et al. used selective antagonists targeting the 5-HT_2A_, 5-HT_2C_, and 5-HT_1A_ receptors to reduce dopamine, 5-HT and glutamate release and WDS response, proposing that 5-HT_2A_ and 5-HT_2C_ receptors are involved in 25I-NBOMe-induced neurotransmitter release and hallucinogenic activity, but no activity related to abuse potential was reported [43]. However, a recent paper of Wojtas and Gołembiowska, indicated that NBOMe drugs act as selective 5-HT_2A_ agonists and that the increase in the neurotransmitters extracellular levels was probably due to the stimulus of the 5-HT_2A_ receptor and subsequent activation of the 5-HT_2C_ receptors [22]. Furthermore, according to Canal and Murnane, “Animal self-administration experiments demonstrate a very strong correlation between drugs that produce dependence in humans and those that are voluntarily consumed by laboratory animals” [62] – and, as observed in Table 7, 25B-NBOMe, 25H-NBOMe, 25D-NBOMe and 2C-P significantly increased the total number of infusions and active lever presses, and 25N-NBOMe and 2C-C significantly increased the total number of infusions, raising awareness on their putative potential for abuse.

In summary, 2C and NBOMe drugs might incite rewarding and reinforcing effects through a dopaminergic mechanism, suggesting their abuse potential. However, further studies are indispensable to fully clarify their effects on dopamine signaling pathways, and to understand how the activation of 5-HT_2C_ receptors can impact the abuse potential of these drugs.

## 4 Conclusions

It is generally accepted that phenylethylamine drugs interact with the neurotransmission systems, where the activation of 5-HT_2A_ receptors acts as a central element in mediating their psychedelic effects. Through a comprehensive analysis of various studies, it becomes clear that the inclusion of an *N*-benzyl moiety considerably enhances the strength of these drugs, particularly the affinity for 5-HT_2A_ receptors. Accordingly, 2C and NBOMe drugs induce behaviors such as WDS, BMC, and HTR, with 5-HT_2A_ receptor antagonists blocking these effects in some cases, thus underscoring the receptor’s role in these responses. Research on these drugs shows the relationship between receptor binding affinity and activation efficacy, highlighting the complex interplay between molecular structure and pharmacological activity. Moreover, the agonist *versus* antagonist properties of these drugs on serotonergic receptors represent another aspect of particular significance, revealing how they affect various paths of communication and different types of receptors. Notably, while some drugs are full agonists in some receptors, others display partial agonist or antagonist effects, suggesting a large spectrum of toxicodynamic actions.

Beyond 5-HT receptors, psychedelic phenethylamines show additional pharmacodynamic effects, including interactions with monoamine oxidases, TAAR_1_, and several monoamine receptors and transporters. Some NBOMe drugs have been shown to increase dopamine levels in key rodent brain regions, despite not directly interacting with dopamine receptors. This effect may be mediated by TAAR_1_ activation, which can modulate dopamine release and reuptake through its influence on other neurotransmitter systems. Notably, while these effects have been demonstrated in rodents, human TAAR_1_ activation remains unproven, underscoring species differences that may impact the drugs' overall effects. This intricate interplay of receptor systems not only shapes their psychoactive properties but also contributes to their abuse potential, a concern increasingly supported by emerging evidence.

Rodents studies have demonstrated that these compounds can induce rewarding and reinforcing effects through dopaminergic mechanisms. Furthermore, alterations in dopamine transmission and related biomarkers in key brain regions associated with addiction support the view of their abuse potential. While additional research is required to better elucidate the mechanisms responsible for their addictive properties, these findings emphasize the need for more studies into the pharmacotoxicological effects of these drugs and putative risks for human health.

## Funding

This work was financed by national funds from FCT - Fundação para a Ciência e a Tecnologia, I.P., in the scope of the project UIDP/04378/2020 and UIDB/04378/2020 of the Research Unit on Applied Molecular Biosciences-UCIBIO and the project LA/P/0140/2020 of the Associate Laboratory Institute for Health and Bioeconomy-i4HB. In addition, this work was also funded by FEDER funds through the Operational Programme Competitiveness Factors-COMPETE and national funds by FCT under research grants UIDB/00081/2020 (CIQUP), LA/P/0056/2020 (IMS) and PT-OPENSCREEN-NORTE-01–0145-FEDER-085468 projects. EGM (SFRH/BD/146527/2019) is supported by an FCT PhD fellowship,  DJB (DL57/2016/CP1355/CT0007) and RS (DL57/2016/CP1346/CT0025) are supported by FCT junior researcher positions.

## CRediT authorship contribution statement

**Daniel José Barbosa:** Writing – review & editing, Conceptualization. **Eva Gil-Martins:** Writing – original draft, Conceptualization. **Renata Silva:** Writing – review & editing, Supervision, Conceptualization. **Fernanda Borges:** Writing – review & editing, Supervision, Conceptualization. **Fernando Remião:** Writing – review & editing, Supervision, Conceptualization.

## Declaration of Competing Interest

The authors declare that they have no known competing financial interests or personal relationships that could have appeared to influence the work reported in this paper.

## Data Availability

No data was used for the research described in the article.

## Footnotes

Appendix A Supplementary data associated with this article can be found in the online version at doi:10.1016/j.toxrep.2025.101890.
