# Supplementary material for: Toxicodynamic insights of 2C and NBOMe drugs – Is there abuse potential?
Source: Toxicol Rep. 2025 Jan 3;14:101890. doi: 10.1016/j.toxrep.2025.101890 (PMC11762925; doi:10.1016/j.toxrep.2025.101890)
Supplement: Supplementary file 1 — Supplementary material [file mmc1.docx]

**Table S1.** Chemical structures of less reported members from the 2C family (2C-R).

|  | | |
| --- | --- | --- |
| 2C-R | Chemical name | **R** |
| 2C-T or 2C-T-1 | 2,5-dimethoxy-4-methylthiophenethylamine | -SCH_3_ |
| 2C-T-3 | 2,5-dimethoxy-4-(methallyl)thiophenethylamine | -SCH_2_CCH_2_CH_3_ |
| 2C-T-16 | 2,5-dimethoxy-4-allylthiophenethylamine | -SCH_2_CHCH_2_ |
| 2C-T-19 | 2,5-dimethoxy-4-tert-butylthio-phenethylamine | -SC(CH_3_)_3_ |
| 2C-T-21 | 2,5-dimethoxy-4-(2-fluoroethylthio)phenethylamine | -SCH_2_CH_2_F |
| 2C-T-21.5 | 2,5-dimethoxy-4-(2,2-difluoroethylthio)phenethylamine | -SCH_2_CHF_2_ |
| 2C-T-22 | 2,5- dimethoxy-4-(2,2,2-trifluoroethylthio)phenethylamine | -SCH_2_CF_3_ |
| 2C-T-25 | 2,5-dimethoxy-4- isobutylthiophenethylamine | -SCH_2_CH(CH_3_)_2_ |
| 2C-T-27 | 2,5-dimethoxy-4- benzylthiophenethylamine | -SCH_2_C_6_H_5_ |
| 2C-T-28 | 2,5-dimethoxy-4-(3-fluoropropylthio)phenethylamine | -S(CH_2_)_3_F |
| 2C-T-30 | 2,5-dimethoxy-4-(4-fluorobutylthio)phenethylamine | -S(CH_2_)_4_F |
| 2C-T-31 | 2,5-dimethoxy-4-(4-trifluoromethylbenzylthio)phenethylamine | -SCH_2_C_7_H_4_F_3_ |
| 2C-T-33 | 2,5- dimethoxy-4-(3-methoxybenzylthio)phenethylamine | -SCH_2_C_6_H_4_OCH_3_ |

**Table S2.** Chemical structures of less reported members from the NBOMe family (25R-NBOMe).

|  | | |
| --- | --- | --- |
| 25R-NBOMe | Chemical name | **R** |
| 25D-NBOMe | 4-methyl-2,5-dimethoxy-*N*-(2-methoxybenzyl)phenethylamine | -CH_3_ |
| 25E-NBOMe | 4-ethyl-2,5-dimethoxy-*N*-(2-methoxybenzyl)phenethylamine | -CH_2_CH_3_ |
| 25H-NBOMe | 2,5-dimethoxy-*N*-(2-methoxybenzyl)phenethylamine | -H |
| 25N-NBOMe | 4-nitro-2,5-dimethoxy-*N*-(2-methoxybenzyl)phenethylamine | -NO_2_ |
